# Efficacy of an external cold and vibrating device in reducing discomfort during the administration of an inferior alveolar nerve block in children: A split-mouth randomised crossover study

**DOI:** 10.12688/f1000research.138340.1

**Published:** 2023-08-15

**Authors:** Ananthu H, Ashwin Rao, Srikant Natarajan, Karuna Yarmunja Mahabala, Anupama Nayak

**Affiliations:** 1 Department of Pediatric and Preventive Dentistry, NITTE (Deemed to be University), AB Shetty Memorial Institute of Dental Sciences (ABSMIDS), Mangalore, Karnataka, India; 2 Department of Pediatric and Preventive Dentistry, Manipal College of Dental Sciences Mangalore, Manipal Academy of Higher Education, Manipal, Karnataka, India; 3 Department of Oral Pathology and Microbiology, Manipal College of Dental Sciences Mangalore, Manipal Academy of Higher Education, Manipal, Karnataka, India

**Keywords:** Pain, Pain management, Analgesia, Local anaesthesia, Dental anaesthesia, Nerve block, Distraction, Paediatric dentistry

## Abstract

**Background:** Local anaesthesia is the backbone of pain management. However, the administration of a local anaesthetic injection itself is considered a painful procedure and triggers fear and anxiety in the patient.

**Methods:** A split-mouth randomised controlled crossover trial was designed to study the efficacy of an “external cold and vibrating” device in reducing discomfort during the administration of an inferior alveolar nerve block (IANB) in children. A total of 40 children who fulfilled the inclusion criteria were evaluated for pain response following administration of IANB with and without the “external cold and vibrating” device. Randomisation was performed to determine whether the subject received the control intervention first or the test intervention first. The intensity of the pain response and discomfort were recorded using the Wong-Baker FACES Pain Rating Scale and the Faces Legs Activity Cry and Consolability scale.

**Results:** There was a statistically significant difference in the pain response between the test group and the control group (p<0.001). Females reported a higher pain response than males, with a statistically significant difference, when the FLACC scores were compared.

**Conclusions:** The “external cold and vibrating” device reduced discomfort during the administration of an IANB in children selected for the study.

## Introduction

Pain management in patients has been one of the most influential factors affecting the success of any dental treatment. In fact, the fear and anxiety surrounding dental procedures has forced patients to resort to a more ‘wait and watch’ approach, delaying or avoiding the dental treatment as much as possible. ^1^ Dental anaesthesia has always played a pivotal role in the management of pain in patients. However, the very procedure inducing anaesthesia, with the local anaesthetic injection, is considered a painful procedure and triggers fear and anxiety in the patient. ^2^ Trypanophobia, or the fear for needles or injections, is considered as one of the major sources of pain in dental patients. ^3^ This clinical problem becomes even more challenging in paediatric patients.

The search for methods to administer a painless dental anaesthetic injection has led to the discovery of various innovative techniques. Several devices and methods have been proposed to tackle this clinical problem, including the use of expensive computer-controlled local anaesthetic delivery systems. ^4^ However, an ideal solution has not been found so far.

One of the techniques that developed over time to help reduce the discomfort during a local anaesthetic injection for dental treatment, is desensitising the injection site, through precooling, employing vibration techniques or a combination of both. ^5^ Distraction is a safe and reliable behaviour management strategy that helps minimise pain and anxiety by directing the attention away from painful stimuli, during the anaesthesia administration. The use of distraction in paediatric patients during local anaesthetic injections, through the application of cold temperature and vibration techniques, has garnered much attention in recent times. ^6^^–^^11^

External cold and vibrating devices propose to reduce pain by altering the individual’s perception to pain. It is hypothesized that the distractive environment created by the vibrating device or buzzing device, coupled with cold, causes the brain cells to relay the non-pain signals (vibration or cold) generated, masking the pain signals triggered by the injection. ^12^^,^^13^ This masking effect of pain is brought about by creating a confusion in the perception of signals by the pain pathway. ^13^^,^^14^ Studies performed have shown the effectiveness of this concept of pain management in immunisations and venepuncture. ^12^^,^^13^

The effectiveness of the “external cold and vibrating” device while administering the maxillary infiltration anaesthesia in children has been reported in literature. ^15^ However, its efficacy to reduce discomfort during the administration of an inferior alveolar nerve block (IANB) in children has not been studied. The aim of the study was to evaluate the efficacy of an “external cold and vibrating device” (Buzzy Mini Personal Striped with ice wings, MMJ Labs, Atlanta, GA, USA), in reducing discomfort during the administration of an IANB in children.

## Methods

### Study design

This randomized controlled clinical trial followed a split mouth, crossover study design with a 1:1 balanced allocation ratio. This study protocol was approved by the Research Ethics Committee of Manipal College of Dental Sciences (MCODS), Mangalore (Reference number 19086) and the trial was registered at the clinical trial registry-India (Registration number CTRI/2020/07/026702). The trial was carried out at the Department of Pediatric and Preventive Dentistry over a period of 23 months. Before enrolment, all participants and their parents/guardians signed written informed consent forms. All procedures were conducted in compliance with the Helsinki Declaration 1975.

### Study subjects

The study was conducted on 40 children who reported to the outpatient department of Pediatric and Preventive Dentistry, MCODS Mangalore, MAHE (Deemed to be University), Mangaluru, Karnataka.

### Sample size calculation

Based on the key article by Alanazi *et al.*, ^15^ the mean and standard deviation of pain response in test and control groups in males was 2 ± 0.32 and 7 ± 0.27 respectively. With 5% alpha error, 90% power of the study and a clinically significant difference of 0.2 units, the required sample in each group was calculated to be 40. The following formula was used to calculate the sample size (N),

$$N=\frac{2{\left(Z_{1-\alpha /2}+Z_{1-\beta }\right)}^{2}\sigma^{2}}{d^{2}}$$

The inclusion criteria for the study were children:

• Aged six to 10 years with no prior experience of an intraoral local anaesthetic injection
• Rated as definitely positive or positive on the Frankl behaviour rating scale
• Classified ASA I according to the American Society of Anaesthesiologists Physical Status Classification System (ASA-PS)
• With any mandibular tooth/teeth indicated for bilateral dental procedures that warranted the use of an IANB.

The exclusion criteria were children with a:

• History of recent hospitalisation or surgery
• History of central nervous system depressants or analgesics consumed within eight hours prior to the treatment
• An existing orofacial oedema, infection, abscess or the presence of systemic comorbidities with special dental treatment considerations.

Written informed assent from the parents and written informed consent from the children were obtained respectively. The control intervention was carried on one side of the patient whereas the test intervention was performed on the contralateral side. The order in which the subjects received the interventions was allocated by randomisation. The right side of the patient received the intervention in the first visit (either control or test) whereas the left side received the intervention in the second visit (either control or test). The wash-out period between the two visits was one week. ^16^

### Randomisation

Randomisation was performed to determine whether the subject received the control intervention first or the test intervention first. This was done by the statistician who was otherwise not involved in the study. A block randomization was done as it helped ensure a 1:1 sample allocation ratio, which meant that equal number of samples were subjected to both the test intervention as well as the control intervention on their first visit. Block randomisation was done using a varied block size of 2 and 4. Allocation concealment was done using opaque envelopes. The block sequences (AB, BA, AABB and so on) was computer-generated, following which the statistician performed random allocation of the samples to the blocks using the random number table. The treatment intervention group codes so generated, ‘A’ or ‘B’, wherein ‘A’ stood for the control intervention (administration of IANB without Buzzy) and ‘B’ stood for the test intervention (administration of IANB with Buzzy), were entered into cards along with the sample number, which was placed in numbered and sequentially arranged envelopes. The cards were wrapped in aluminium foil to confirm that the envelopes appeared opaque. The envelopes were sealed. In this way, the predictability of the sample allocation which is the disadvantage of block randomization was overcome. The envelopes were opened just prior to the introduction of the interventions, to avoid bias. ^17^

The treatment protocol described in the envelope, was carried out on the right side of the patient while the alternative treatment was done on the contralateral side in the subsequent visit.

### Interventions

The site of needle penetration was topically anesthetized with 20% benzocaine topical anaesthetic gel (‘Precaine B, Pascal International, Bellevue, WA, USA’) four minutes prior to the injection in both the groups. The children assigned to the control group were administered the IANB with a conventional technique. ^18^ The contents of the entire cartridge containing 1.8 mL of 2% lignocaine with 1:80,000 adrenaline was injected ^19^ (Septodont Healthcare India Pvt. Ltd, Maharashtra, India).

**Figure 1.**
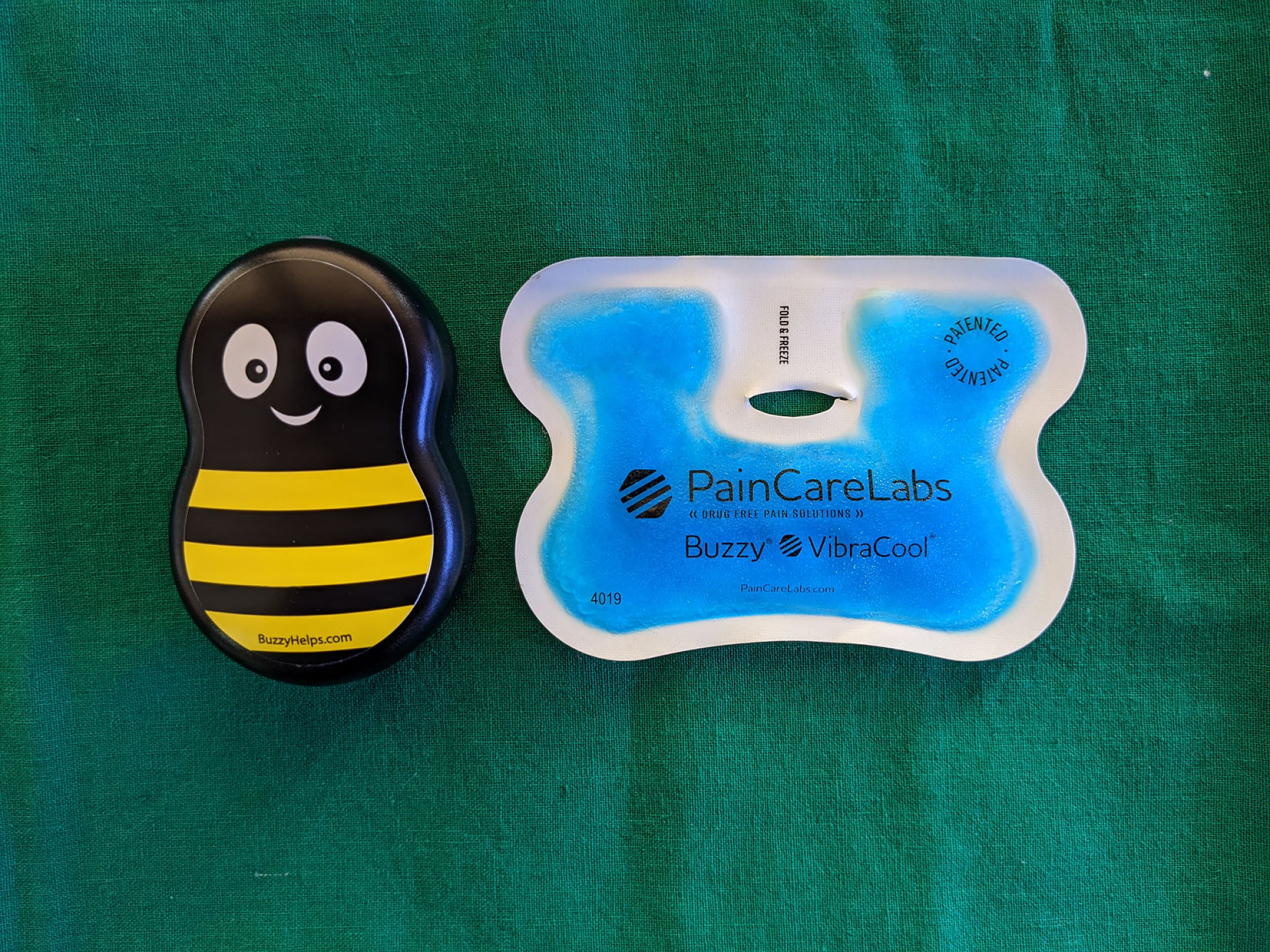
External cold and vibrating device (Buzzy Mini with VibraCool Wings, MMJ labs, Atlanta, GA, USA).

**Figure 2.**
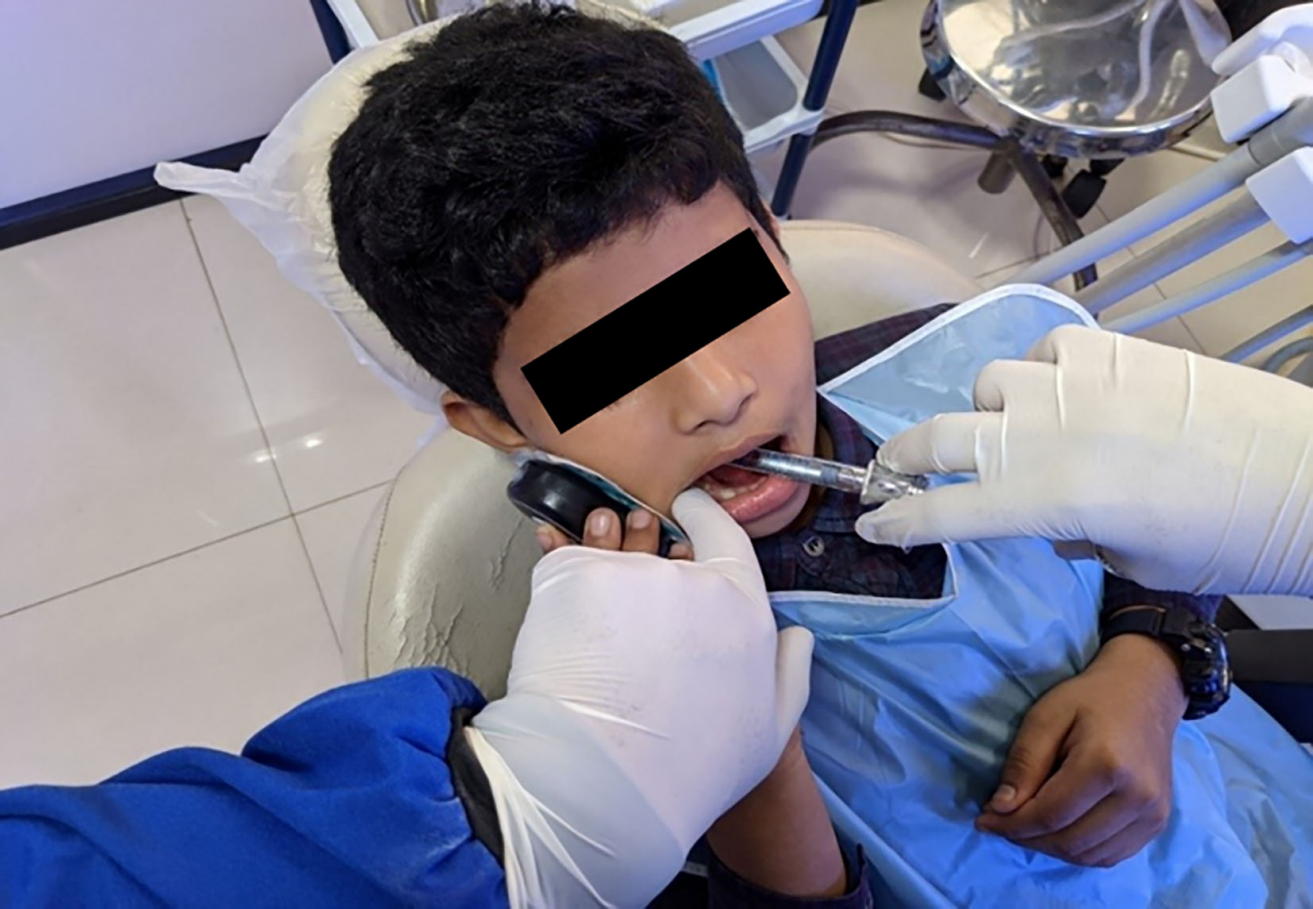
IANB administration coupled with the Buzzy device.

The children assigned to the test group were administered the IANB coupled with the “external cold and vibrating” device (Buzzy Mini Personal Striped with Ice Wings, MMJ labs, Atlanta, GA, USA). The Buzzy ( Figure 1) is a bee-shaped device which offers vibrations, powered by two alkaline AAA batteries and cold temperature offered by the detachable, refrigerable ice wings. ^20^ Prior to the delivery of anaesthesia by injection, the refrigerated wings were attached to the bee shaped body of the device, following which the child was asked to hold and keep the device on the cheek area, corresponding to the ramus of the mandible ( Figure 2). After thirty seconds, the administration of IANB was initiated, with the Buzzy device switched on manually. The device was switched off, upon completion of the administration of the anaesthetic solution.

Each child was instructed to quantify the pain experienced during the injection using the “Wong-Baker Faces Pain Rating Scale” (WBFPRS) following administration of the IANB. The WBFPRS is a horizontal scale of six hand-drawn faces, scored from 0 to 10, that range from a smiling ‘no hurt’ face on the left to a crying ‘hurts worst’ face on the right. It is simple to use and implement, and it is well accepted by children, parents, and doctors. ^21^ However, it being a self- reported scale, the child’s report of pain may be affected by developmental, cognitive and situational issues. ^22^ To overcome this drawback, another scale was used as an adjunct to this scale. This was the Faces Legs Activity Cry and Consolability (FLACC) scale, which assesses five different aspects of the child’s behaviour. This scale is shown to have good validity and reliability. The five categories are assessed in order to rate pain wherein each category is ranked on a three-point scale (0-2), resulting in a summary score of (0-10). ^23^^,^^24^ An independent precalibrated evaluator, rated the children on the FLACC scale during the injection.

### Statistical analysis

The Statistical Package for Social Science (SPSS) (version 20.0) software package was used to analyse the data. The comparison between responses of the participants to pain on the WBFPRS at the test visit and control visit and the observed behaviour of participants using the FLACC scale at the test visit and control visit was measured by Wilcoxon signed rank test. The level of significance was set at 5% ( *i.e.* <0.05).

## Results

In total, 40 children aged six to 10 years, with any mandibular tooth/teeth indicated for bilateral dental procedures that warrants the use of an IANB, were recruited for the study. The 40 recruited children comprised 13 (32.5%) females and 27 (67.5%) males with a mean age of 7.90 years (S.D ± 1.533). The percentage of males recruited for the study were almost double that of females.

### Comparison of pain response during administration of the IANB in the control group and test group ( Table 1)

**Table 1.** Comparison of pain response between (i) Administration of IANB without Buzzy ^®^ (control group) and Administration of IANB with Buzzy ^®^ (test group) using Wilcoxon signed rank test.

		N	Mean±SD	Median (IQR)	Range (Min-Max)	z value	p value
F	WBFPRS control	13	3.54±1.45	4(2,4)	2 to 6	-3.127	**0.002**
	WBFPRS test	13	1.54±0.88	2(2,2)	0 to 2		
F	FLACC control	13	4.38±0.77	5(4,5)	3 to 5	-3.256	**0.001**
	FLACC test	13	1.69±0.75	2(1,2)	1 to 3		
M	WBFPRS control	27	3.19±1.39	2(2,4)	2 to 6	-4.866	**<0.001**
	WBFPRS test	27	0.81±1	0(0,2)	0 to 2		
M	FLACC control	27	3.63±0.84	3(3,4)	3 to 6	-4.678	**<0.001**
	FLACC test	27	1.15±0.72	1(1,1)	0 to 3		
Total	WBFPRS control	40	3.3±1.4	4(2,4)	2 to 6	-5.767	**<0.001**
	WBFPRS test	40	1.05±1.01	2(0,2)	0 to 2		
Total	FLACC control	40	3.88±0.88	4(3,5)	3 to 6	-5.655	**<0.001**
	FLACC test	40	1.32±0.76	1(1,2)	0 to 3		

F = Females, M = Males.

The p-values have been mentioned in bold and have been underlined to denote that the values are statistically significant which is very vital for the results of the study.

• Among females, on comparison of the median score values of WBFPRS control and WBFPRS test, the median score value of WBFPRS control was higher than that of the WBFPRS test by two units with a p-value of 0.002 (statistically significant).
• Among females, on comparison of the median score values of FLACC control and FLACC test, the median score value of FLACC control was higher than that of test by three units with a p-value of 0.001 (statistically significant).
• Among males, on comparison of the median score values of WBFPRS control and WBFPRS test, the median score value of WBFPRS control was higher than that of the WBFPRS test by two units with a p-value <0.001 (statistically significant).
• Among males, on comparison of the median score values of FLACC control and FLACC test, the median score value of FLACC control is higher than that of test by two units with a p-value <0.001 (statistically significant).
• Analysing the 40 participants, the median score value of WBFPRS control was higher than that of test by two units with a standard score (Z value) of -5.767. The WBFPRS score comparison gave a p-value <0.001 which was statistically significant.
• Analysing the 40 participants, the median score value of FLACC control was higher than that of the test by three units with a standard score (Z value) of -5.655. The FLACC score comparison gave a p-value <0.001 which is statistically significant.

The inference from the above statements was that pain scores were higher for both the WBFPRS and FLACC scales in the control group.

## Discussion

The results of the present study showed that the experimental Buzzy device demonstrated a statistically significant effect in reducing self-reported and observer-reported reaction to pain during the administration of the IANB. These findings support the results of previous studies, which had reported success with the combined use of cold and vibration to alleviate discomfort in children undergoing maxillary infiltration dental anaesthesia. ^15^

The Buzzy is an economical, versatile, quickly vibrating plastic device designed like a bee, which combines high-frequency vibration and cold temperature to control sharp pain. The pain control mechanism is on the basis of the “gate control” pain relief theory by confusing the body’s own nerves, thereby dulling or eliminating sharp pain. The brain closes the gate on pain signals when nerves receive non-painful signals such as vibration or cold. Another mechanism by which the Buzzy device claims its effect is through “diffuse noxious inhibitory control” or DNIC wherein certain noxious signals are dampened out by the brain. The intense cold also activates a supraspinal modulation raising the body’s overall pain threshold. ^20^^,^^25^

As pain and discomfort are subjective and vary from child to child, it’s difficult to compare the effectiveness of one approach to another when employed separately in different children. ^14^ To avoid such variances, the split-mouth cross-over design was employed, which has been used in other trials investigating the effectiveness of the Buzzy device. ^9^^,^^10^ Crossover trials are most appropriate for studies related to pain as well as when the treatment effects are reversible and short-lived. ^26^

Randomisation was performed to determine whether the subject would receive the control intervention first or the test intervention first. A block randomization was done as it helped ensure a 1:1 sample allocation ratio, which meant that equal number of samples were subjected to both the test intervention as well as the control intervention on their first visit.

This study included children aged six to 10 years old, as this has been suggested as the age at which cognitive growth begins to reveal itself. ^27^ This will ensure adequate communication between the operator and the child for the purposes of the study. Inal *et al*. in 2012 ^28^ and Moadad *et al*. in 2016 ^29^ recruited a similar age group in their studies.

In the present study, two separate pain scales were used to ensure the reproducibility of the pain assessment.

Although subjective pain assessment is typically deemed the gold standard for assessing pain, children’s perceptions of pain might well be exaggerated, especially soon after the treatment. Pain is a highly complex and multidimensional attribute that depends on various factors such as location, quality of sensory perception and the cognitive ability of the child. Use of only a subjective scale may not reflect the true pain and discomfort experienced by the child patient. Hence observational pain scales should be used in conjunction with subjective scales. For pain evaluation in young children, the FLACC scale has been proven to showcase great validity and reliability. Hence, in this study both self-report WBFPRS and the observational FLACC scale was used.

This study selected only children rated definitely positive and positive on the Frankl rating scale. This was done to negate the effect of behaviour on the results of the study. Lack of blinding, which could not be carried out due to the nature of the intervention, was also a shortcoming of this trial

Future studies can include anxious children or children of a younger age group to study the effects of the external cold and vibrating device.

## Conclusions

The results showed that there was a statistically significant difference in the pain response when the control group and the test group were compared (p<0.001). Based on the inferences from the current study it can be concluded that the “external cold and vibrating” device reduced the discomfort during the administration of an IANB in children selected for the study.

### Future directions

Research trials should be conducted in the future to explore the effectiveness of the “external cold and vibrating” device during the various different techniques of local anaesthesia administration and among children belonging to different age groups.

## Authors’ contributions

All the authors have equal contribution to this research in manuscript preparation, data collection and interpretation.

## Data Availability

Figshare: Master data for the control group and the test group.xlsx, https://doi.org/10.6084/m9.figshare.23498491.v1. ^30^ This project contains the underlying following data: Data file 1: Master data for the control group and the test group Data file 2: Results table Data file 3: Sample size calculation Figshare: Extended data, https://doi.org/10.6084/m9.figshare.23498508.v1. ^31^ This project contains the underlying following data: Data file 1: Child assent form Data file 2: Clinical trial protocol Data file 3: FLACC Scale Data file 4: IEC Approval Thesis Data file 5: Informed consent sheet Data file 6: Pain Rating Scale Data file 7: Patient information sheet Figshare: CONSORT check list and flow chart for “Efficacy of an External Cold and Vibrating Device in Reducing Discomfort during the Administration of an Inferior Alveolar Nerve Block in Children: A Split Mouth Randomised Crossover Study”, DOI: https://doi.org/10.6084/m9.figshare.23498673.v1. ^32^ Data are available under the terms of the Creative Commons Attribution 4.0 International license (CC-BY 4.0).
