# Supplementary material for: Efficacy of an external cold and vibrating device in reducing discomfort during the administration of an inferior alveolar nerve block in children: A split-mouth randomised crossover study
Source: F1000Res. 2023 Aug 15;12:984. [Version 1] doi: 10.12688/f1000research.138340.1 (PMC10973762; doi:10.12688/f1000research.138340.1)
Supplement: Peer review history

# Reviewer report: Reviewer response for version 1

**DOI:** 10.5256/f1000research.151530.r197889

**Published:** 2024-03-27

**Authors:** Kavitha Ramar

**Affiliations:** 1 Pediatric and Preventive Dentistry, SRM Kattankulathur Dental College, SRM Institute of Science & Technology, Chennai, Tamil Nadu, India

This clinical trial addresses one of the most important challenges faced by dentists around the globe - managing pediatric dental patients and it has done justice in validating an excellent practical solution. The research article has been well presented, and is scripted with detailed descriptions under all the sections. 

 The Title of the study “Efficacy of an external cold and vibrating device in reducing discomfort during the administration of an inferior alveolar nerve block in children: A split-mouth randomised crossover study” is simple, understandable and outlines the theme of the research in a nutshell. 

 The Abstract is precise, well-structured and has been summarized adequately. 

 The Introduction provides an excellent opening to the theme of the research wherein the challenges faced by pediatric dentists and the techniques they employ to manage pediatric dental patients while administering local anaesthesia have been clearly outlined. The external cold and vibrating device Buzzy is a novel armamentarium which has been utilised by doctors and other healthcare givers while managing pediatric patients, however the limited research in the field of dentistry has triggered the researchers to come up with this proposed study, which they have substantiated with proper evidence.

 The authors have succeeded in clearly chalking out the necessary armamentarium utilised for the research. A step wise methodology has been presented, which would help fellow researchers who wish to pursue their research further in the same arena. A randomised clinical trial is evaluated by how comprehensibly the randomisation process has been described and followed. The authors have succeeded in this aspect by outlining the randomisation process in a very systematic manner. The split mouth design of the study has also been justified. Thus, the selection of the study design is justified and the work showcases academic merit.

 The results and interpretations are sound and supported by relevant and current evidence. The authors have stated the findings, highlighting key aspects in tables and figures. Commenting on the statistical analysis, by employing the Wilcoxon test, the authors have helped understand the study results better.

 The Discussion part is excellently structured and has been elucidated in a very appropriate manner justifying all the results and findings of the study. The discussion of the findings is suitable, gives answers to the research objectives, incorporating substantial support from literature. Cross references with similar as well as contrasting research have been appropriately described by the authors, which give a scope of better understanding for fellow researchers.  

 The authors have even mentioned the drawbacks of the trial, which is appreciable.  One of the drawbacks of the study is the selection of only cooperative children which has also been mentioned by the authors. By mentioning the limitations of the study, authors have hinted at the scope for the improvement of the study. 

 The conclusions are sound, justified and logically explained, and are supported by the results. No revisions are required.

Is the work clearly and accurately presented and does it cite the current literature?

Yes

If applicable, is the statistical analysis and its interpretation appropriate?

Yes

Are all the source data underlying the results available to ensure full reproducibility?

Yes

Is the study design appropriate and is the work technically sound?

Yes

Are the conclusions drawn adequately supported by the results?

Yes

Are sufficient details of methods and analysis provided to allow replication by others?

Yes

Reviewer Expertise:

Nano dentistry, special children,  behaviour management

I confirm that I have read this submission and believe that I have an appropriate level of expertise to confirm that it is of an acceptable scientific standard.

# Reviewer report: Reviewer response for version 1

**DOI:** 10.5256/f1000research.151530.r229569

**Published:** 2023-12-28

**Authors:** Meenu Mittal

**Affiliations:** 1 ESIC Dental College & Hospital, Delhi, India

It's a good research but some details/corrections are required:

 Methods:

 1.Correction regarding Assent/consent required.

 2. Intervention- It is not mentioned why the injection was given and which procedure was performed post injection for treatment of which tooth.

 Results:

 1.Needs to be compiled, written haphazardly with text matter repeated.

 Discussion:

 1.The description about pain scales etc. to be shortened

 2. There is no comparison with other studies discussed, which needs to be added.

 Limitations: To be elaborated.

 Language: Small corrections in grammar needed.

Is the work clearly and accurately presented and does it cite the current literature?

Yes

If applicable, is the statistical analysis and its interpretation appropriate?

I cannot comment. A qualified statistician is required.

Are all the source data underlying the results available to ensure full reproducibility?

No source data required

Is the study design appropriate and is the work technically sound?

Yes

Are the conclusions drawn adequately supported by the results?

Yes

Are sufficient details of methods and analysis provided to allow replication by others?

Yes

Reviewer Expertise:

Local anesthesia in pediatric patients

I confirm that I have read this submission and believe that I have an appropriate level of expertise to confirm that it is of an acceptable scientific standard, however I have significant reservations, as outlined above.

# Reviewer report: Reviewer response for version 1

**DOI:** 10.5256/f1000research.151530.r197890

**Published:** 2023-09-04

**Authors:** Vivek Padmanabhan

**Affiliations:** 1 RAK Medical and Health Sciences University, Ras Al-Khaimah, United Arab Emirates

Good research and may help to resolve this ambiguous part of pediatric dentistry, however the practical applicability remains to be seen. 

 Please note that the entire article has been split into a lot of paragraphs. Kindly revise it. 

 The part where it is mentioned consent and assent was taken please note that assents are taken from children and consent is taken from adults, so correct it accordingly.

 In the statistical analysis, it has been mentioned Wilcoxon has been used, kindly elaborate how this test helps understand the study better, also give complete details of the SPSS as in brand name and associated details. 

 In the discussion please compare the results of your study with other studies done worldwide and then either defend the findings or agree with others. 

 The first sentence of conclusion is not necessary. in conclusion also mention about differences between males and females as I believe that was one of your objectives.

 The future recommendations need to be provided based upon the limitations of the present study also please note that the sample number should also be increased in future studies, this can be added as both drawback and address it in future recommendations. 

 Please note that the references can be kept within a maximum of 10 years else it is not clinically significant for today and need not be cited in the present study.

Is the work clearly and accurately presented and does it cite the current literature?

Partly

If applicable, is the statistical analysis and its interpretation appropriate?

I cannot comment. A qualified statistician is required.

Are all the source data underlying the results available to ensure full reproducibility?

Yes

Is the study design appropriate and is the work technically sound?

Yes

Are the conclusions drawn adequately supported by the results?

Yes

Are sufficient details of methods and analysis provided to allow replication by others?

Yes

Reviewer Expertise:

Pediatric Dentistry, Dentistry, Epidemiological studies

I confirm that I have read this submission and believe that I have an appropriate level of expertise to confirm that it is of an acceptable scientific standard, however I have significant reservations, as outlined above.
